# A Postnatal Critical Period for Orientation Plasticity in the Cat Visual Cortex

**DOI:** 10.1371/journal.pone.0005380

**Published:** 2009-04-29

**Authors:** Shigeru Tanaka, Toshiki Tani, Jérôme Ribot, Kazunori O'Hashi, Kazuyuki Imamura

**Affiliations:** Laboratory for Visual Neurocomputing, RIKEN Brain Science Institute, Wako, Saitama, Japan; L'université Pierre et Marie Curie, France

## Abstract

Orientation selectivity of primary visual cortical neurons is an important requisite for shape perception. Although numerous studies have been previously devoted to a question of how orientation selectivity is established and elaborated in early life, how the susceptibility of orientation plasticity to visual experience changes in time remains unclear. In the present study, we showed a postnatal sensitive period profile for the modifiability of orientation selectivity in the visual cortex of kittens reared with head-mounted goggles for stable single-orientation exposure. When goggle rearing (GR) started at P16-P30, 2 weeks of GR induced a marked over-representation of the exposed orientation, and 2 more weeks of GR consolidated the altered orientation maps. GR that started later than P50, in turn, induced the under-representation of the exposed orientation. Orientation plasticity in the most sensitive period was markedly suppressed by cortical infusion of NMDAR antagonist. The present study reveals that the plasticity and consolidation of orientation selectivity in an early life are dynamically regulated in an experience-dependent manner.

## Introduction

Neuronal circuits in the mammalian visual cortex maintain certain dynamic mechanisms of structural and functional modification in the early postnatal period as well as in adulthood. Typically, monocular deprivation in early postnatal days leads to a shift in the dominant responses of primary visual cortical neurons to the non-deprived, experienced eye [1]–[4]. A deficit of vision caused by retinal damage is compensated for by rewiring of cortical circuits even in adult animals [5]. A different type of plasticity has been found with regard to the orientation selectivity of visual cortical neurons: it is currently thought that the orientation selectively is innately generated [6], and then maintained [7] and elaborated [8] by visual experience in early postnatal days. Experimentally, this orientation plasticity is induced by exposure of young animals to retinal images restricted to a single orientation [9]–[16]. However, results to date are variable, reflecting the various methods used for inducing and detecting the orientation plasticity. This variability has made it difficult to delineate the postnatal critical period during which orientation plasticity manifests.

Recent technological progress in optical imaging of intrinsic signals [17]–[21] and data processing methods for noise reduction [22], [23] now provide an efficient method, complementary to conventional unit recording, for revealing overall features of the cortical distribution of orientation selectivity. In addition, head-mounted cylindrical-lens-fitted goggles [24] provide an efficient tool for restricting visual experience to a single orientation. We previously reported that attaching goggles to a kitten continuously without interruption by dark-rearing episodes was particularly effective in causing modification of orientation selectivity [15].

In this study, we used these technical improvements to determine the critical period for orientation plasticity in cat primary visual cortex. It has been shown that infusion of an NMDA receptor antagonist to the visual cortex suppresses the expression of orientation selectivity [25], [26]. It is further expected that NMDA-receptor-mediated long-term potentiation (LTP) at synapses to visual cortical neurons optimally activated by the exposed orientation is involved in the over-representation of that orientation. We confirmed that cortical infusion of an NMDA receptor antagonist nearly abolished the modifiability of orientation selectivity during the critical period. We also addressed an important question of how the orientation map modified by single-orientation exposure is consolidated afterwards.

## Results

### Normal rearing

**Figure 1:**
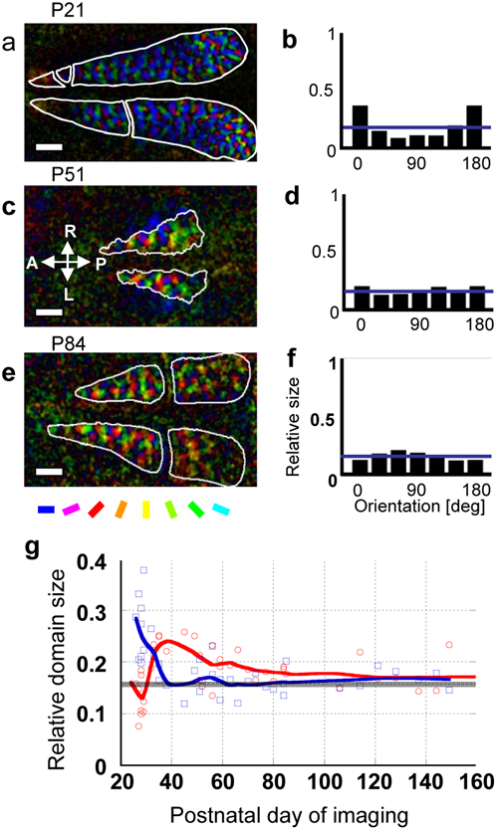
Orientation selectivity in normally reared kittens. Data are shown for postnatal days indicated. (a), (c) and (e): orientation polar maps. Color and brightness indicate preferred orientation and orientation selectivity. White curves delineate functionally defined area 17. Scale bars, 2 mm. Color code is placed below e. (b), (d) and (f): orientation histograms. Height of bins indicates the relative size of cortical domains preferentially responding to stimulus orientations. Horizontal lines indicate the relative size of iso-orientation domains for a uniform orientation representation. The same bin is doubly depicted at 0° and 180° for symmetric representation. Cortical coordinate placed on c: A, anterior; P, posterior; R, right; and L, left. (g): the relative size of cortical domains preferentially responding to vertical orientation (red dots) and horizontal orientation (blue dots) plotted against the postnatal day of optical imaging repeated in 23 normally reared kittens. Two curves were obtained using Stineman's smoothing procedure.

Figure 1 shows three orientation polar maps (Figs. 1a, c and e) and corresponding orientation histograms (Figs. 1b, d and f), obtained from normally reared kittens of different ages. At P29, the relative size of responsive domains is largest for horizontal orientation (0° or equivalently 180°) and smallest for vertical orientation (90°) (Fig. 1b). The sample-averaged orientation histogram across 10 normally reared kittens in the age range of P26–32 exhibited a bias toward horizontal orientation (Fig. S1a online). A one-way repeated-measures ANOVA on the relative sizes of cortical domains for 6 stimulus orientations supports this observation (p = 0.0001). The relative size of domains representing 0° was significantly larger than that of the other orientations, except for 150° (p<0.0001 for 60° and 90°; p = 0.0002 for 120°; p = 0.0005 for 30°; ANOVA, post-hoc analysis by Tukey's HSD test). This indicates that orientation representation is biased toward the horizontal orientation for very young normal kittens. This is analogous to the innate bias toward the contralateral eye, as has been known in ocular dominance [27].

The horizontal bias diminished at P51 (Fig. 1d), and shifted to a weak vertical bias at P84 (Fig. 1f). The sample-averaged orientation histogram across 15 normally reared kittens of P33–84 showed a weak bias toward vertical orientation (Fig. S1b online). A one-way repeated measures ANOVA on the relative sizes of cortical domains indicates non-uniform orientation distribution (p = 0.0001). The following pairs showed significant differences: 90° and 30°, 90° and 150°, and 90° and 0° (p = 0.0011, p<0.0001 and p = 0.014, respectively; post hoc analysis by Tukey's HSD test), but the other pairs did not show significant differences.

Figure 1g shows the relative domain size for 90° (red open circles) and for 0° (blue open squares) repeatedly measured in 23 normally reared kittens, as a function of the postnatal day of optical imaging. The red and blue curves were obtained by a smoothing procedure to show slight but significant age-dependent changes of orientation selectivity in normal kittens; the horizontal bias was prominent before P35, but thereafter it was taken over by the vertical bias.

### Goggle rearing

**Figure 2:**
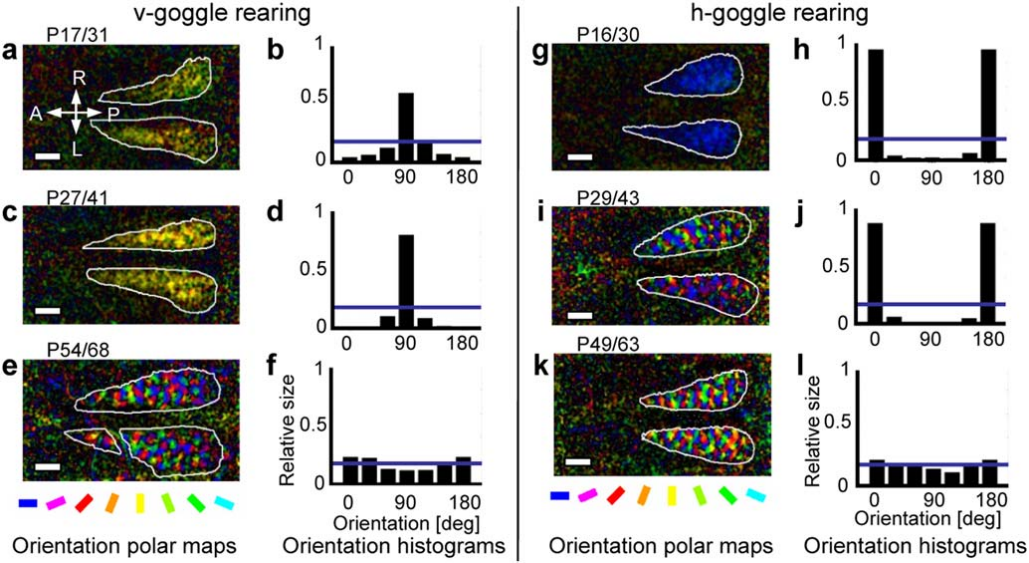
Orientation selectivity in kittens continuously exposed to vertical- or horizontal orientation for 2 weeks. Data obtained by starting/ending GR at the indicated postnatal days are illustrated similarly to Fig. 1a–f.

Here, we first describe the results of two weeks of goggle rearing (GR). Measurements in goggle-reared kittens were completed within 5 hrs after the removal of the goggles. Immediately after the 2-week GR started at P16–P17, the over-representation of horizontal orientation was exclusive (Figs. 2g and h), whereas that of vertical orientation was somewhat moderate (Figs. 2a and b). For GR that started at P27–P29, the over-representation of vertical orientation also became nearly exclusive (Figs. 2c and d). The representation of horizontal orientation was still predominant, although that of unexposed orientations appeared (Figs. 2i and j). The imbalance between vertical and horizontal orientations at P16–P17 may reflect, at least partly, the horizontal bias detected in normal kittens (Figs. 1a and b). When 2-week GR was started at P49–54, the induced over-representation of the exposed orientation, either vertical or horizontal, diminished drastically (Figs. 2e, f, k and l). A close examination of Figs. 2e and f revealed that the effect of vertical GR was reversed to a slight under-representation of the vertical orientation.

**Figure 3:**
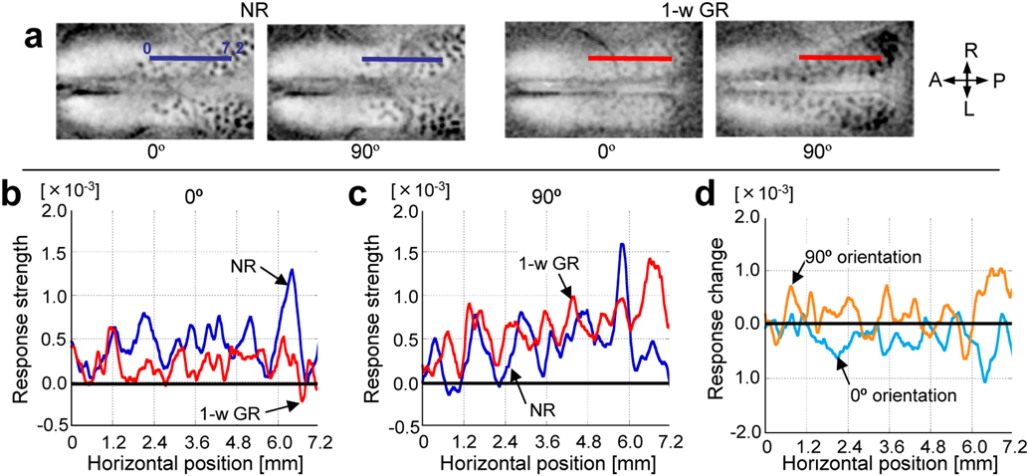
Comparison of orientation selectivity before and after GR. (a): single-orientation maps representing responses to 0° and 90°, constructed at P28 in a normally reared kitten (NR) and at P38 immediately after 1-week vertical-GR in the same animal (1-w GR). Darkness in the maps indicates the strength of responses to stimulus orientations. Horizontal lines drawn inside functionally defined area 17 are superimposed on the maps and placed on the corresponding cortical position. Length of the lines, 7.2 mm. (b): profiles of response strengths for 0° before and after GR along the horizontal lines (blue: before; red: after). (c): those for 90°. (d): profiles of changes in response strengths through GR (differences between the two curves in b and c). Orange: 90°; sky blue: 0°. We carefully calibrated the baseline level of responses so that the response strength averaged over the range of the 2.4-mm extension of the horizontal lines into functionally defined area 18, either before or after GR. Stimulus gratings of a 0.5 c.p.d. spatial frequency presently used do not evoke area 18 (see Materials and Methods).

Figure 3 shows which changes in the orientation representation during 1-w GR also sufficiently modify orientation selectivity if adequately timed. In Fig. 3a, single-orientation maps of a kitten at P28 after normal rearing show a patchy organization for stimulus orientations (0° and 90°). After 1-w vertical GR from P31 to P38 in the same kitten, patchy organization became more prominent for the exposed orientation (90°), and the domain size increased particularly around the posterior part of the visual cortex. In contrast, patchy organization for the unexposed orientation (0°) almost disappeared. To quantify these changes, profiles of response strengths were measured along the line (7.2 mm long) drawn anteroposteriorly in the single-orientation maps (0°: Fig. 3b; 90°: Fig. 3c) and compared between the data from before and after GR. Figure 3d depicts the net decrease or increase of response strengths caused by GR. Our results show that vertical GR decreases response strengths at 0° by −1.5×10^−4^±1.2×10^−5^ (s.e.) per pixel on average (Figs. 3b and d), whereas those at 90° increase by 1.1×10^−4^±1.5×10^−5^ (s.e.) (Figs. 3c and d). This implies that, in local response strengths, the suppression occurring for the unexposed orientation is comparable with or even larger than the enhancement occurring for the exposed orientation. Such suppression and enhancement (Fig. 3d) together account for the marked over-representation of the exposed orientation, observed in the orientation polar maps (Figs. 2a, c, g and i).

### Critical period of orientation selectivity

**Figure 4:**
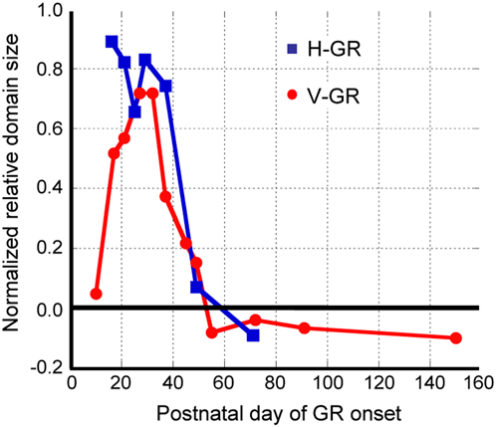
Sensitivity profiles for orientation plasticity. Ordinate: orientation sensitivity defined by (*x*-*y*)/(1-*y*), where *x* represents the relative size of cortical domains for the exposed orientation in a goggle-reared kitten, and *y* represents the value of the smoothed curves for normal kittens (Fig. 1g) for the same orientation. Red dots: vertical orientation; blue squares: horizontal orientation. Abscissa: postnatal day of 2-week GR onset.

Figure 4 illustrates the time profile of the sensitivity for the modification of orientation selectivity as revealed by 2-week GR in 18 kittens. Here, we quantified the sensitivity as the relative size of the cortical domains for the exposed orientation, which is normalized by the representation bias in normal kittens (Fig. 1g) at respective days of optical imaging (see legend of Fig. 4). The sharp enhancement obtained for vertical- and horizontal-orientation exposures are generally consistent with each other, except for a discrepancy at 2–3 postnatal weeks. Taken together with the horizontal bias observed in normal kittens as mentioned above, this discrepancy in the sensitivity profiles may suggest that the innate bias toward horizontal orientation is inherent to neuronal mechanisms that determine orientation selectivity. The critical period can be defined as the postnatal period during which 2-week GR applied (started and/or ended) effectively causes the modification of orientation selectivity. Thus defined, the critical period starts 2 weeks after birth and lasts for 6 weeks.

Figure 4 also shows that the critical period is followed by a late phase of under-representation of the exposed orientation for GR starting between P55 and P151. We also observed the case of an adult cat reared with vertical goggles from P396 for one month; this cat showed a small relative size of cortical domains representing the exposed orientation (0.11) for 90°. Therefore, continuous single-orientation exposure after the critical period, even in adulthood, leads to the under-representation of the exposed orientation.

### NMDA receptor antagonist blocks modifiability of orientation selectivity

To test whether NMDA receptors contribute to the critical period of orientation selectivity, we infused the left visual cortex with an NMDA receptor antagonist, D-(-)-2-Amino-5-phosphonopentanoic acid (D-AP5), by an osmotic minipump system [28]. In three kittens, we carried out 1-week concurrent GR and minipump infusion.

**Figure 5:**
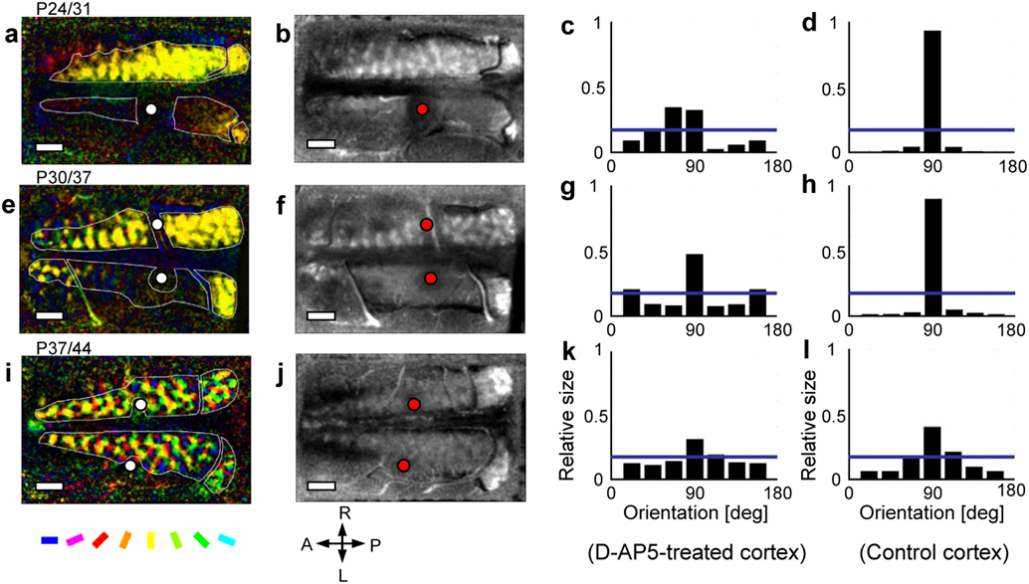
Effect of NMDA receptor antagonist on orientation plasticity. (a), (e) and (i): orientation polar maps constructed immediately after concurrent GR and D-AP5 infusion for 1 week in three kittens for different onset ages of GR. (b), (f) and (j): response-strength maps constructed by summing all single-orientation maps in the respective animals. Brightness indicates response strength. White dots in a, e and i, and red dots in b, f, and j indicate locations of infusion centers of D-AP5 in the left-hemisphere or saline in the right hemisphere. (c), (g) and (k), and (d), (h) and (l) show orientation histograms for the D-AP5-treated visual cortex and for the control visual cortex, respectively. Other conventions are as in Fig. 1.

In the kitten starting GR and D-AP5-infusion at P24, the darkness in the orientation polar map in the D-AP5-infused hemisphere (Fig. 5a lower) indicates an overall reduction of orientation selectivity. This contrasts with the almost full representation of the exposed vertical orientation in the non-infused (control) right hemisphere (Fig. 5a upper). The response-strength maps in Figs. 5b, f and j show that, although visual responses completely disappeared within the diameter of 1–2 mm from the site of D-AP5-infusion (red dots), low levels of visual responses were preserved outside of this unresponsive region. In orientation histograms, the D-AP5-infused hemisphere showed a weak over-representation of vertical orientation (Fig. 5c), as contrasted to the virtually full representation in the control hemisphere (Fig. 5d). Similar results were obtained in the kitten with GR and D-AP5 infusion starting at P30 (Figs. 5e–h). In the other kitten started at P37 at the late critical period (Fig. 4), D-AP5 was virtually ineffective (Figs. 5i–l).

**Figure 6:**
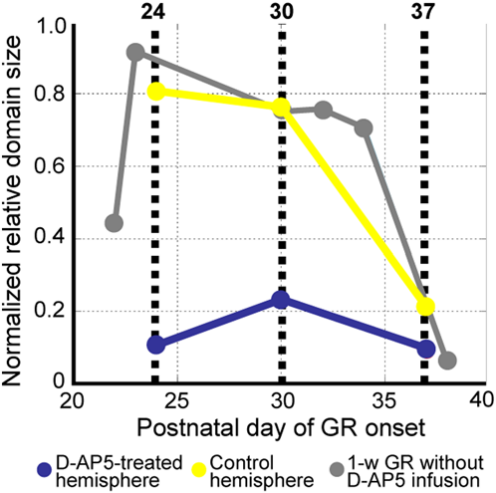
Effects of D-AP5 infusion on the sensitivity profile. Sensitivity profiles are shown for control and D-AP5-treated hemispheres, separately. For reference, we plot the sensitivity profile for 1-week GR without D-AP5 infusion obtained from 6 kittens. Symbols are defined in the figure.

Figure 6 illustrates the sensitivity profile of orientation selectivity for 1-week GR. The critical period defined for 1-week GR (grey dots) rapidly ended before P40, earlier than the end of the critical period defined for 2-week GR (Fig. 4). The ineffectiveness of D-AP5 infusion at P37 on the induction of over-representation of the exposed orientation seems consistent with the end of the critical period for 1-week GR. The sensitivity was drastically suppressed in the D-AP5-infused hemispheres of kittens goggle-reared from P24 and P30 (blue dots), whereas the D-AP5-untreated hemispheres still exhibited large sensitivity (yellow dots) comparable to that for goggle-reared kittens without D-AP5 infusion (grey dots).

### Orientation selectivity modification under short- and long-term GR

**Figure 7:**
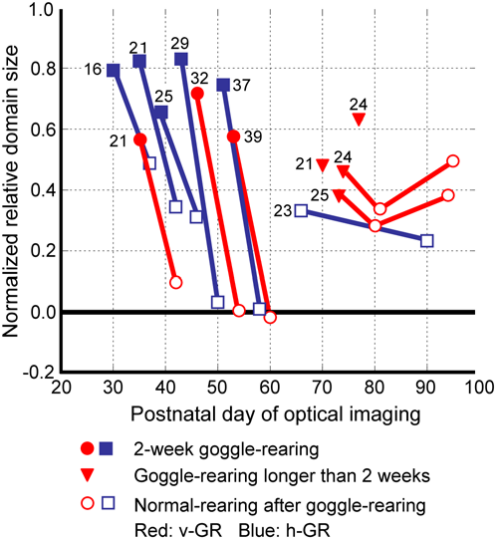
Effects of duration and timing of GR. The relative size of cortical domains preferentially responding to the exposed orientation is plotted against the postnatal day of optical imaging. Symbols are indicated in the figure. Plots obtained from identical kittens are linked by solid lines. Numerical figures indicate onset ages of GR.

In Fig. 7, we illustrate how the once-induced over-representation of the exposed orientation changes afterwards. On the left half of the diagram, the normalized relative sizes of cortical domains representing exposed vertical (circles) or horizontal (squares) orientations are plotted at the end of 2-week GR (solid symbols) and also at the end of the succeeding normal rearing (hollow symbols), respectively. Plotted points for identical kittens are linked by the lines. In 4 kittens for which 2-week GR started at P29, 32, 37 and 39, 3-day normal vision immediately before the second optical imaging eliminated the over-representation; the normalized relative domain sizes returned to 0 (level of normal kittens). However, in 4 kittens in which 2-week GR started relatively earlier at P16, P21 and P25, recovery during the succeeding 3-days of normal vision was partial.

On the right half of the diagram, triangles plot for 4–6 week GR. In 4 kittens with long-term vertical GR started at P21–P25 (triangles), the over-representation of the exposed orientation was retained at moderate levels between 0.39 and 0.64 in the first optical imaging. As tested in two of these kittens (P24–P73; P25–P74), the over-representation was preserved even after 3 weeks of normal rearing. A similar tendency was observed in another kitten in which horizontal GR started at P23 and switched to normal rearing at P51. To summarize, recovery of GR-induced changes are time-dependent in threefold: 1) when 2-week GR covers the relatively late phase of the critical period (at P29–39), normal rearing quickly eliminates the over-representation; 2) when 2-week GR covers the relatively early phase of the critical period (earlier than P29), the diminution of the over-representation is moderate; and 3) GR for 4 weeks or more outlasting the critical period acts to preserve the moderate over-representation of the exposed orientation.

## Discussion

In this study, we applied a combination of head-fixed cylindrical-lens goggles and optical imaging of intrinsic signals to investigate postnatal orientation plasticity in kitten primary visual cortex. By measuring the over-representation of exposed orientation using optical imaging at the end of 1- or 2-week GR, we revealed a brief period exhibiting a sharp increase in the sensitivity of orientation modifiability between postnatal 3–5 weeks (Fig. 4 and Fig. 6). This increase was nearly abolished after 1-week infusion of D-AP5 (Fig. 6). The presently delineated critical period for orientation plasticity overlaps the most sensitive period for ocular dominance plasticity (postnatal 4–5 weeks) [29]. It may be worth mentioning that the method presently applied to kittens would have broad application to various animal species. We recently reported an over-representation of the exposed orientation in goggle-reared young rats [30], in which individual neurons showing orientation selectivity are distributed in the visual cortex without grouping in the form of orientation maps.

### NMDA-dependent orientation plasticity

Neuronal mechanisms underlying the increased responses to the exposed orientation have yet to be investigated, but based upon the wealth of knowledge about cortical synaptic plasticity so far accumulated, the following conjecture may be made. In cortical synapses, activity-dependent LTP could be induced at synapses with postsynaptic NMDA receptors by the coincidence of pre- and postsynaptic activities [31]. Such LTP could occur at those synapses to visual cortical neurons optimally activated by the exposed orientation. The NMDA receptor dependence of the GR-induced over-representation of exposed orientation (Figs. 5 and 6) supports this view.

The NMDA receptor is composed of obligatory NR1 subunits and other NR2 subunits such as NR2A and NR2B. Because D-AP5 effectively blocks NMDA receptors containing NR2A subunits more than those containing NR2B subunits [32], it may be that NR2A subunits contribute more to GR-induced modification of orientation selectivity than NR2B subunits. Indeed, it has been suggested that signaling through an NR2A-PSD95 complex is important for the maturation of orientation selectivity by visual experience in mice [33]. It has also been shown that activation of NR2A-containing, but not NR2B-containing, NMDA receptors, induces activity-dependent dendritic protein synthesis for synaptic changes in cultured hippocampal neurons [34]. Because NR2A subunits are persistently expressed in the superficial layers from postnatal 1 week up to adulthood in cats [35], the brief age-dependence of orientation modification may require a gating mechanism of the downstream signaling of NR2A-containing NMDA receptors to determine a basic structure of orientation maps through LTP induction associated with dendritic protein synthesis.

### NMDA-independent orientation plasticity

The decreased responses to unexposed orientations for GR, as shown in Fig. 3, are also one of the major causes of the over-representation of the exposed orientation [11]. Close examination to the D-AP5-infused kittens reveals that, in the control hemisphere, response strengths to the unexposed orientations 0°, 30° or 150° are comparable to those to any orientations in the D-AP5-infused hemisphere (Fig. S2 online). This observation suggests that the decrease of responses to unexposed orientations does not involve NMDA receptor function and possibly is mediated by a process such as NMDA-independent LTD. One possible mechanism of the reduction of responses to unexposed orientations may be activity-dependent LTD induced by the activation of cannabinoid receptors located at presynaptic sites [36], [37], but the elucidation of the mechanism requires further investigation.

### Modifiability of orientation selectivity after the critical period

The presently revealed brief critical period for orientation modifiability (Fig. 4) contrasts with the long tail phase of ocular dominance plasticity [29]. This discrepancy suggests different neural mechanisms of ocular dominance and orientation plasticity. In this regard, it may be worth noting that the over-representation of the exposed orientation shifts over to under-representation after the critical period for orientation plasticity (Fig. 4). There may be opposing forces that expand and contract the area of cortical domains representing the exposed orientation in early life. If it is the case, the end of the critical period may be determined by the age at which the contraction dominates the expansion, resulting in the rapid decrease of the sensitivity profile for orientation plasticity different from the long tail of the sensitivity profile for ocular dominance plasticity.

In accordance with the above-mentioned under-representation of the exposed orientation, decreased neuronal responses to the exposed orientation have been shown in unit recording experiments. An early experiment on adult cats performed by Creutzfeldt and Heggelund revealed a decrease in the number of neurons optimally responding to vertical orientation exposed in a striped environment for 1 hr, twice a day during 2 weeks [38]. As speculated by these authors, adaptation-dependent response modification may be a mechanism of orientation plasticity after the end of the critical period. A recent experiment has shown a similar form of orientation-dependent adaptation in the orientation tuning curves of neurons, which rapidly changed in adult cats when one orientation was presented more frequently than the other orientations [39]. However, the time scale of such adaptation is distinct in the order of that of the under-representation observed in the present study. A question remains on whether common mechanisms of adaptive response reduction work in wide range of time scales.

On the other hand, it has been reported that intracortical microstimulation induced the expansion of an iso-orientation domain around the stimulation site in adult cats [40]. In addition, the pairing of single-orientation visual stimulation and the injection of depolarizing current to visual cortical neurons shifted preferred orientations towards the exposed orientation in adult cats as well as in kittens [41]. These findings seem to be contradictory to the under-representation of the exposed orientation observed in the present study. Considering that our goggle-reared cats after the end of the critical period simply experienced a single orientation in the freely moving condition, the forced stimulation to the cortex may uncover the suppression of synaptic plasticity that would be latent in the adult visual cortex.

### Persistence of modified orientation selectivity

Goggle rearing for 1 or 2 weeks modifies orientation preferences to nearly match the exposed single orientation, but such changes quickly diminish upon returning kittens to normal rearing (Fig. 7). Extension of GR for 4 weeks or longer is required for obtaining a persistent over-representation of exposed orientation, which was preserved at least 4 weeks after the kittens were returned to normal vision (Fig. 7).

Very recently, Ohzawa et al. (SFN Abstract 2007-A-109015) performed single-unit recordings in cats that were reared with our goggles for vertical orientation exposure. The persistent, modest over-representation of the exposed orientation observed after long-term GR is supported by recordings using the subspace reverse-correlation-mapping technique [42]. They have shown that a substantially increased number of neurons in cat areas 17 and 18 responded optimally to the exposed orientation after long-term (>45 days) GR started at P21–28. This effect was robust, irrespective of the length of normal rearing intervals (0–50 days) before single-unit recording.

### Comparisons with previous studies

There have been two hypotheses about the effect of visual experience on orientation plasticity. One is the selection hypothesis and the other the instruction hypothesis. In the former hypothesis, after single-orientation exposure, neurons innately selective for unexposed orientations just decrease their responses to the unexposed orientations without changes of preferred orientations. In the latter hypothesis, neurons selective for unexposed orientations change their preferred orientations towards the exposed orientation. Blakemore and Cooper [9] supported the instruction hypothesis, because they found preferred orientations of recorded units were strongly biased toward the exposed orientation in single-unit recording in cats that had experienced striped environment. Later, Stryker et al. [11] found in cats that had experienced parallel lines with opaque goggles [10] that single-orientation exposure changed a large portion of units nonselective or unresponsive, although responsive units preferring for the exposed orientation relatively increased. Particularly, they observed an orderly arrangement of selective units according to preferred orientation along the electrode tracks, as found in normal cats, but clustering of nonselective or unresponsive units frequently appeared.

In our optical imaging on kittens exposed to a single orientation, stimulus-related intrinsic signals in response to unexposed orientations were reduced in cortical domains originally selective for the unexposed orientations (Fig. 3b), and a proportion of pixels without orientation selectivity tended to increase [15], consistently with single-unit recording by Stryker et al. [11]. However, stimulus-related intrinsic signals in response to the exposed orientation tended to increase in these domains (Figs. 3c and d), resulting in the changes of orientation preference. As seen in Fig. 5C of Stryker et al. [11], it seems that nonselective/unresponsive units were rather distributed randomly, even if they might be weekly clustered along the track. In addition, this figure showed a wide range of a constant preferred orientation around the exposed horizontal orientation from 3–6 mm in the track distance. Therefore, this figure does not definitely support that neurons innately selective for unexposed orientations decrease selectivity or responsiveness without changes of their preferred orientations.

In the single-unit recordings by Ohzawa et al. (SFN Abstract 2007-A-109015) on cats reared with our goggles for vertical orientation exposure, when they penetrated electrodes into the medial bank, they also recorded responsive units in these cats fewer than in normal cats, as in Stryker et al. [11]. Among the responsive units, most units were selective for the exposed orientation, but intriguingly the number of units selective for the orthogonal horizontal orientation was larger than that of units selective for oblique orientations (Fig. S3 online). The presence of the orthogonally oriented units may not be accounted for by the selection hypothesis alone, because horizontally oriented units should be most unlikely detected if the lack of experience decreases selectivity or responsiveness. On the contrary, in the instruction hypothesis, preferred orientations closer to the exposed orientation may be shifted more effectively towards the exposed orientation, and the orthogonally oriented units can possibly remain with their preferred orientations unchanged due to the largest separation in the preferred orientation from the exposed orientation, as we have previously postulated a possible mechanism for orientation modification (Fig. 8 in [15]).

Differences of experienced patterns during single-orientation exposure may be worth noting. Stryker et al. [11] exposed kittens to stationary lines through their goggles. Carlson et al. [16] also presented stationary stripe patterns with various spatial frequencies to monocularly deprived infant monkeys. To examine the effect of exposure to stationary oriented stimuli, we have tried to rear 4 kittens chronically with spherical-lens-fitted goggles for exposure to a stationary stripe with a spatial frequency of about 0.5 and 0.15 c. p. d [24]. Although the exposed orientation was over-represented at the first optical imaging experiments after 2- or 3-week GR in 3 kittens, the under-representation of the exposed orientation occurred in the other kitten, in which the orthogonal orientation was over-represented (Fig. S4 online). Even in the kittens showing the over-representation of the exposed orientation, the layouts of orientation preferences were labile during prolonged GR. In 3 of the 4 kittens, the over-representation disappeared or changed to the under-representation after long-term GR. Such labile alteration of orientation maps is characteristic of exposure to a stationary oriented pattern. This is contrasted with the finding that orientation maps altered by exposure to a dynamic single orientation through cylindrical-lens-fitted goggles are consolidated preserving the modest over-representation of the exposed orientation (Fig. 7). It should be noted that the instability in orientation map alteration for stationary stripe pattern exposure was not due to the repeated optical imaging, because orientation maps altered by rearing with cylindrical-lens-fitted goggles changed gradually in each optical imaging, and were finally stabilized at the moderate over-representation of the exposed orientation. The fact that Carlson et al. [16] recorded units selective for the orthogonal orientation to the exposed orientation in the open eye may be such labile modification of orientation selectivity induced by the stationary stripe pattern exposure. The disappearance of the orientation selectivity modification for prolonged exposure to stationary oriented stimuli is suggested to have a weak impact on the structural modification of orientation maps. This may be consistent with the fact that Stryker et al. [11] observed gradual and progressive changes of the preferred orientation of selective units along the electrode track in kittens exposed to stationary lines, which are similar to those observed in normal cats.

### Conclusion

Orientation plasticity appears to have two phases: First, the visual cortical circuit is rendered highly modifiable in orientation selectivity during a brief postnatal critical period for 6 weeks; and second, modified orientation selectivity consolidates during continuous long-term single-orientation exposure. The combined application of GR and optical imaging to not only cats but also rodents would be instrumental to further analyses of molecular and cellular mechanisms underlying this unique system phenomenon, orientation plasticity.

## Materials and Methods

### Preparation of animals

We used 58 kittens (normal: 23; goggle-reared: 35) obtained from the institutional colony. The procedures of surgery and optical imaging were approved by the Institutional Animal Research Committee at RIKEN (No. H13-B040), and performed in accordance with the “Guiding Principles for the Care and Use of Animals in the Field of Physiological Science” of the Japanese Physiological Society. We performed chronic optical imaging repeatedly twice or three times for 12 among 35 goggle-reared kittens to see the recovery or consolidation of altered orientation maps, and for 15 among 23 normally reared kittens to see the age-dependent changes of orientation representation bias. We made efforts to minimize the animals' suffering and to reduce the number of animals examined as much as possible. We refrained from performing optical imaging experiments within 7 days after surgery, and from repeating optical imaging on identical animals at an interval shorter than 7 days. After experiments, we sacrificed animals injecting the overdose of pentobarbital (50–100 mg/kg, i.v.).

### Goggles for single-orientation exposure

We used goggles fitted with planoconvex acrylic cylindrical lenses (lens thickness, 10.0 mm; lens aperture diameter, 15.0 mm; lens power, +67 D), through which the animals were able to see elongated images of their environments [24]. We used two types of goggles: v- and h-goggles, which elongated visual images vertically and horizontally, respectively.

### Surgical procedure

Surgery was conducted according the procedure described in our previous papers [14], [15]. Initial anesthesia was induced using ketamine hydrochloride (5.0 mg/kg, i.m.) following sedation with medetomidine hydrochloride (0.1 mg/kg, i.m.). The animals were fixed on a stereotaxic apparatus and were artificially ventilated with a 60∶40% mixture of N_2_O and O_2_ containing 0.5–1.0% isoflurane. Heart rate, end-tidal CO_2_ concentration, and rectal temperature were continuously monitored during surgery. A metal head holder for fixing the goggles and a metal chamber for optical imaging were cemented on the animal's skull using dental resin, and the skull and dura mater covering the recording area of the lateral gyrus were removed. The cranial window (17 mm×12 mm) was positioned approximately from P5 to A12, spanning the midline. Next, the chamber was filled with 2% agar and sealed with a polyvinylidene chloride thin film and a plastic plate. Finally, the frame of the goggles was fixed to a head holder and the position of the goggles was calibrated so that the cylindrical lenses covered the visual field as widely as possible.

### Optical imaging

Animals were anesthetized as in surgery and paralyzed with pancuronium bromide (0.1 mg/kg/h). They were artificially ventilated. Contact lenses with appropriate curvatures were used to prevent the drying of eyes. The cortex was illuminated with a 700-nm wavelength light. The focal plane was adjusted to 500 µm below the cortical surface using a tandem-lens macroscope arrangement [18]. Intrinsic optical signals were measured while the animals were exposed to visual stimuli displayed on a 20-inch CRT monitor placed 30 cm in front of the animal. Images were obtained with a CCD video camera, and digitized and stored using CAPOS (320×240 pixels) [43] or Imager 3001 (744×480 pixels) (Optical imaging Inc. New York). For each stimulus presentation, the intrinsic signal was recorded for 1.0 s before and 5.0 s after the stimulus onset. A blank stimulus was presented for 15 s between successive captures of intrinsic signals. Each visual stimulus was presented once in a pseudorandom sequence in a single trial of recordings. Twenty-six to 30 trials were collected in each recording session. As visual stimuli, we used full-screen square-wave gratings, which drifted in two directions at six equally spaced orientations (interval, 30°). To functionally identify area 17, we used gratings of a spatial frequency of 0.5 c.p.d., which is optimal for area 17 neurons [20], [44], [45]. The temporal frequency of the gratings was fixed at 2.0 Hz. The optical imaging in one session was completed within 5 hours.

### Cortical infusion of D-AP5

During 1-week GR, D-AP5 was continuously infused into the visual cortex of 3 kittens using an osmotic minipump system. The method has been described in detail elsewhere [46]. In brief, D-AP5 (Tocris Cookson Inc.) was prepared, using sonication, as a 50-mM solution with sterilized distilled water or PBS (pH 7.4). We made a cannula of 33 G hypodermic needle, connected to an osmotic minipump that contained D-AP5 solution, and inserted the cannula stereotaxically at a depth of 1.0 mm around AP0, aiming at the border of areas 17 and 18. They were fixed on the skull with dental cement. The infusion rate was 1 µl/hr for a duration of 1 week. In two kittens, the right visual cortex was infused with saline as control. In optical imaging, we used stimulus gratings of a 0.15 c.p.d. spatial frequency for activating both areas 17 and 18 [20], [45], because D-AP5 spread widely over the lateral gyrus including the two areas.

### Analysis of optical imaging data

The analysis methods that we used were described in a previous paper [15]. It is noteworthy to explain the methods in some detail here to show that observed map changes are attributable to biological changes rather than artificial changes originating from our analysis methods. One trial of optical imaging was composed of six frames (duration of each frame, 1 s). To extract stimulus-related intrinsic signals, we subtracted signals recorded in the first frame (without stimulus presentation) from those signals recorded in succeeding frames with stimulus presentations. Then, we averaged the subtracted signals over the 4th to 6th frames for each trial. Next, we applied the generalized indicator function method to these averaged signals [22], which efficiently excluded noisy signals originating from volume and oxygenation changes in thick blood vessels and spatially slowly varying fluctuations of signals inherent in the recorded intrinsic signals. It should be noted that the image data processing based on the generalized indicator function method underestimates the effects of over-representation of exposed orientation induced by single-orientation exposure, because the data processing method eliminates spatially slowly varying point-spread components of intrinsic signal [21], which may partially contain responses to the exposed orientation.

Having excluded the spatially slow noise components, we summed the stimulus-related signals over all trials for each stimulus orientation and applied Gaussian low-pass filtering with a 150-µm standard deviation to eliminate high-frequency noise. In this way, we constructed a *single-orientation map* for each stimulus orientation. We also defined an *integrated response-strength map* by summing single-orientation maps over all stimulus orientations.

To determine the preferred orientation at each pixel inside the recorded area, we used the vector sum method [19], which is based on the Fourier analysis in the circular symmetric orientation dimension. Thus, at each pixel, we obtained the preferred orientation and the modulation amplitude in the second harmonic components, which is regarded as orientation selectivity. The *orientation polar map* was constructed with the preferred orientation and orientation selectivity as color and brightness, respectively.

For further analysis, we discarded pixels eliciting response strengths lower than a half of the response strength averaged over all pixels inside the recorded area. According to this criterion, the domains containing the remaining pixels nearly lined up with functionally defined area 17, which was exclusively activated by stimuli of a 0.5-c.p.d. spatial frequency. To construct an *orientation histogram*, we counted the number of pixels involved in each orientation, 30° width, and normalized them by the total number of pixels involved in all orientations.

## Supporting Information

Figure S1 Sample-averaged orientation histograms of normal kittens. (a, b): Orientation histograms obtained by averaging 10 normal kittens (P26–32), and 15 normal kittens (P45–84), respectively. *: p<0.05; **: p<0.005; ***: p<0.001 (post hoc analysis by Tukey's HSD test). In this analysis, we used data obtained from repeated optical imaging on 9 animals of the age of P26–32 and 6 animals of the age of P33–84 as different samples. Innate orientation bias changes in the early stage of postnatal development. To investigate GR-induced alteration of orientation representation, it is important to examine such innate changes in normally reared kittens. Preferred orientation is significantly biased toward horizontal orientation at P26–32 (a). However, this bias tends to change toward weak but significant vertical-orientation bias (b).
(3.83 MB TIF)

Figure S2 Effects of D-AP5 infusion in single-orientation maps. Single-orientation maps of a kitten, in which 1-week GR and concurrent D-AP5 infusion were started at P24 and P30, respectively. Corresponding cortical domains in the right control hemisphere are delineated by blue lines and those in the left D-AP5-treated hemisphere are delineated by red lines. The observation that response strength to the unexposed orientation decreases during GR (Fig. 3) raises the question of whether NMDA receptor activation is involved in this response reduction. To answer this question, we used single-orientation maps of the kitten in which 1-week GR concurrent with D-AP5 infusion into the left hemisphere started at P 24. Responses to any orientations were weak, as seen inside of the regions delineated by red lines. Note, however, that these responses in the D-AP5-infused left hemisphere are of about the same intensity as the responses to unexposed orientations 0°, 30° and 150° in the right hemisphere (enclosed by blue lines). These findings indicate that the blockade of NMDA receptors does not affect the decline of response strengths to unexposed orientations. Hence, it is unlikely that an NMDA receptor is involved in the reduction of responses to unexposed orientations.
(4.48 MB TIF)

Figure S3 Orientation histogram obtained from single-unit recordings along the tracks when electrodes were penetrated into the medial bank of 3 cats exposed to vertical orientation. By courtesy of Dr. Ohzawa.
(2.06 MB TIF)

Figure S4 Time course of the normalized relative area of the exposed orientation in kittens exposed to a stationary stripe pattern. Blue: horizontally oriented stripe of 0.15 cpd; Red: vertically oriented strip of 0.15 cpd; Sky blue: horizontally oriented stripe of 0.5 cpd; Pink: vertically oriented stripe of 0.5 cpd. The origin in the day of GR indicates the onset day when GR was started.
(5.47 MB TIF)
