# Supplementary figures and images for: A Postnatal Critical Period for Orientation Plasticity in the Cat Visual Cortex
Source: PLoS One. 2009 Apr 29;4(4):e5380. doi: 10.1371/journal.pone.0005380 (PMC2671604; doi:10.1371/journal.pone.0005380)

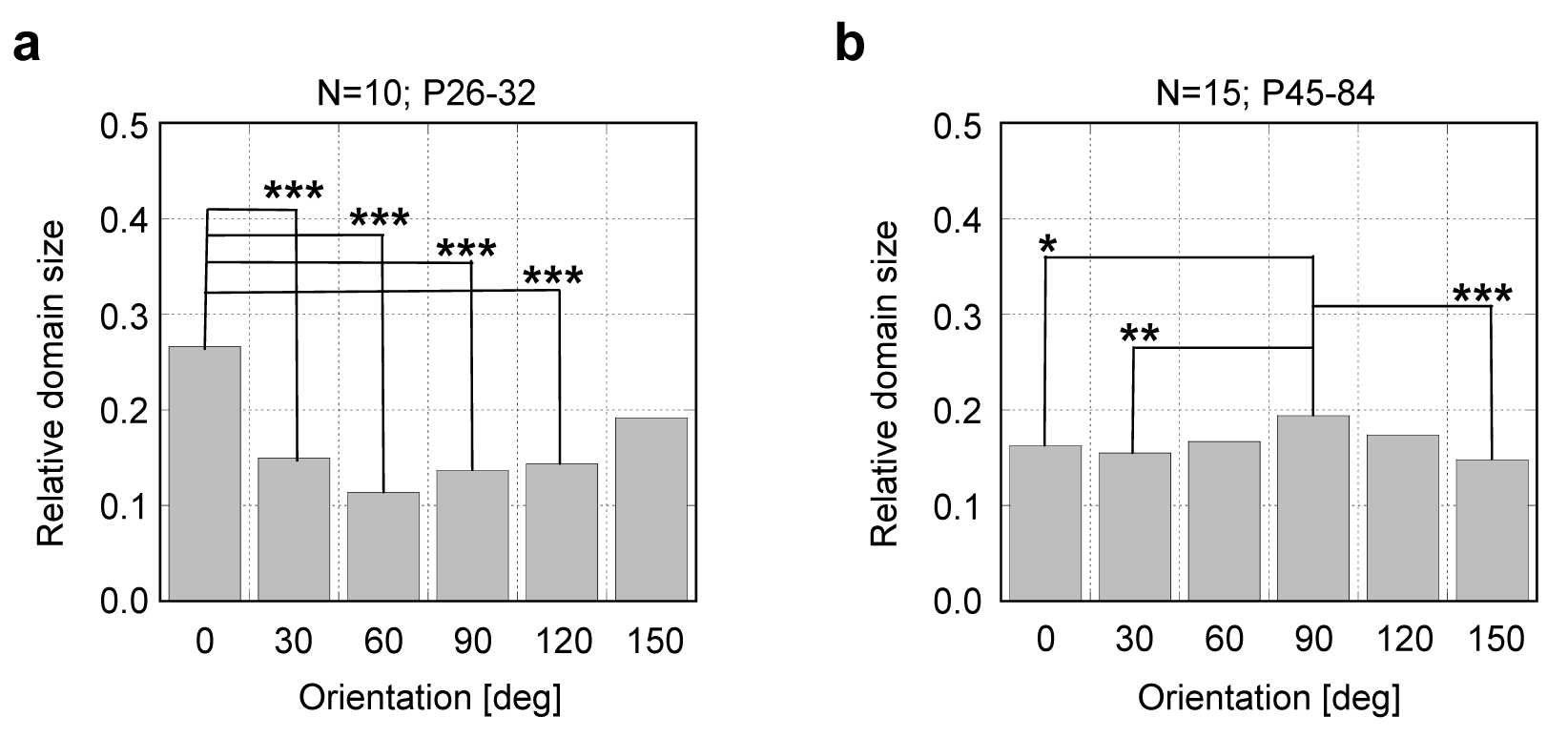

Supplement: Figure S1 — Sample-averaged orientation histograms of normal kittens. (a, b): Orientation histograms obtained by averaging 10 normal kittens (P26–32), and 15 normal kittens (P45–84), respectively. *: p<0.05; **: p<0.005; ***: p<0.001 (post hoc analysis by Tukey's HSD test). In this analysis, we used data obtained from repeated optical imaging on 9 animals of the age of P26–32 and 6 animals of the age of P33–84 as different samples. Innate orientation bias changes in the early stage of postnatal development. To investigate GR-induced alteration of orientation representation, it is important to examine such innate changes in normally reared kittens. Preferred orientation is significantly biased toward horizontal orientation at P26–32 (a). However, this bias tends to change toward weak but significant vertical-orientation bias (b). (3.83 MB TIF) [file pone.0005380.s001.tif]

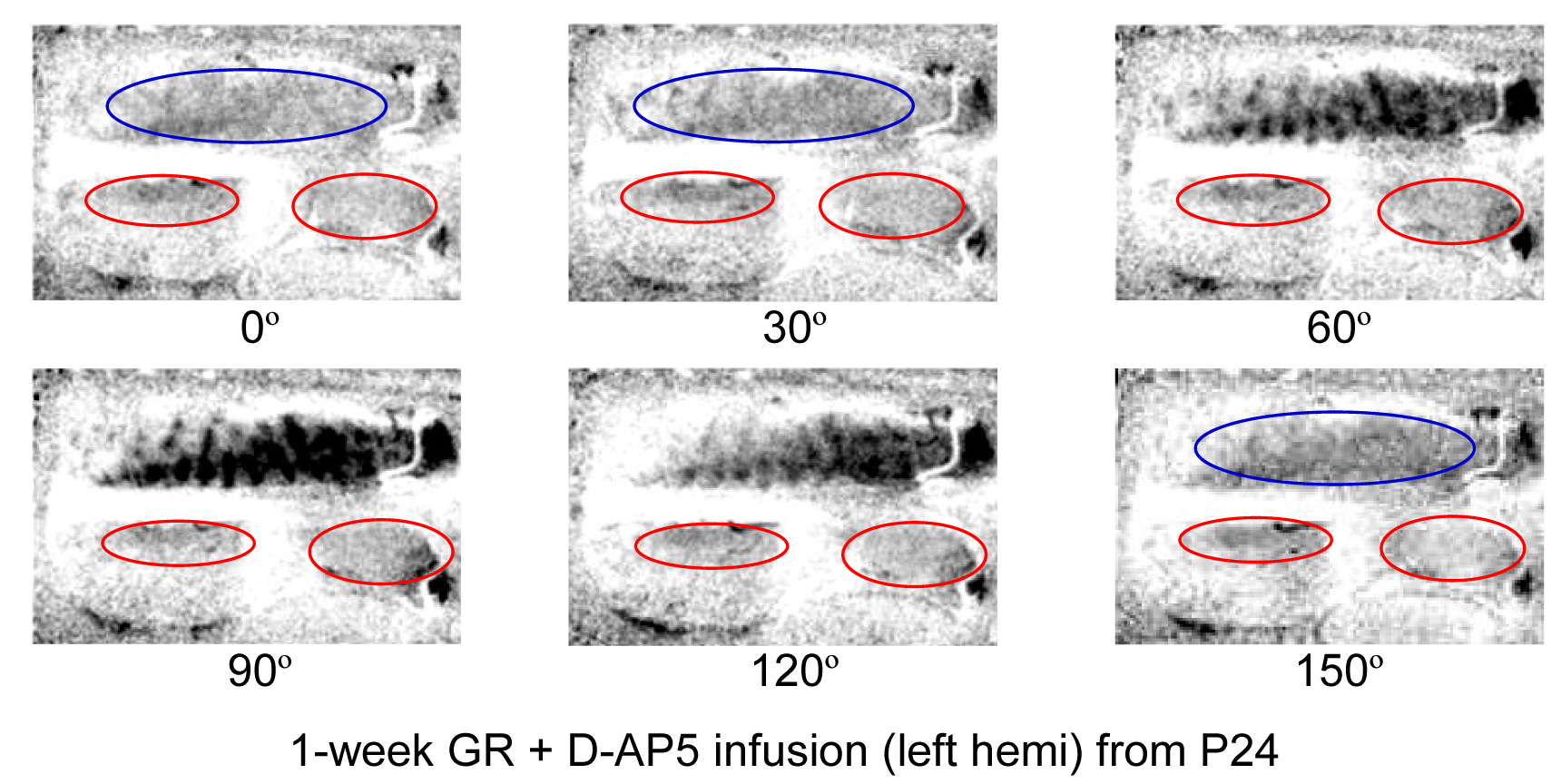

Supplement: Figure S2 — Effects of D-AP5 infusion in single-orientation maps. Single-orientation maps of a kitten, in which 1-week GR and concurrent D-AP5 infusion were started at P24 and P30, respectively. Corresponding cortical domains in the right control hemisphere are delineated by blue lines and those in the left D-AP5-treated hemisphere are delineated by red lines. The observation that response strength to the unexposed orientation decreases during GR (Fig. 3) raises the question of whether NMDA receptor activation is involved in this response reduction. To answer this question, we used single-orientation maps of the kitten in which 1-week GR concurrent with D-AP5 infusion into the left hemisphere started at P 24. Responses to any orientations were weak, as seen inside of the regions delineated by red lines. Note, however, that these responses in the D-AP5-infused left hemisphere are of about the same intensity as the responses to unexposed orientations 0°, 30° and 150° in the right hemisphere (enclosed by blue lines). These findings indicate that the blockade of NMDA receptors does not affect the decline of response strengths to unexposed orientations. Hence, it is unlikely that an NMDA receptor is involved in the reduction of responses to unexposed orientations. (4.48 MB TIF) [file pone.0005380.s002.tif]

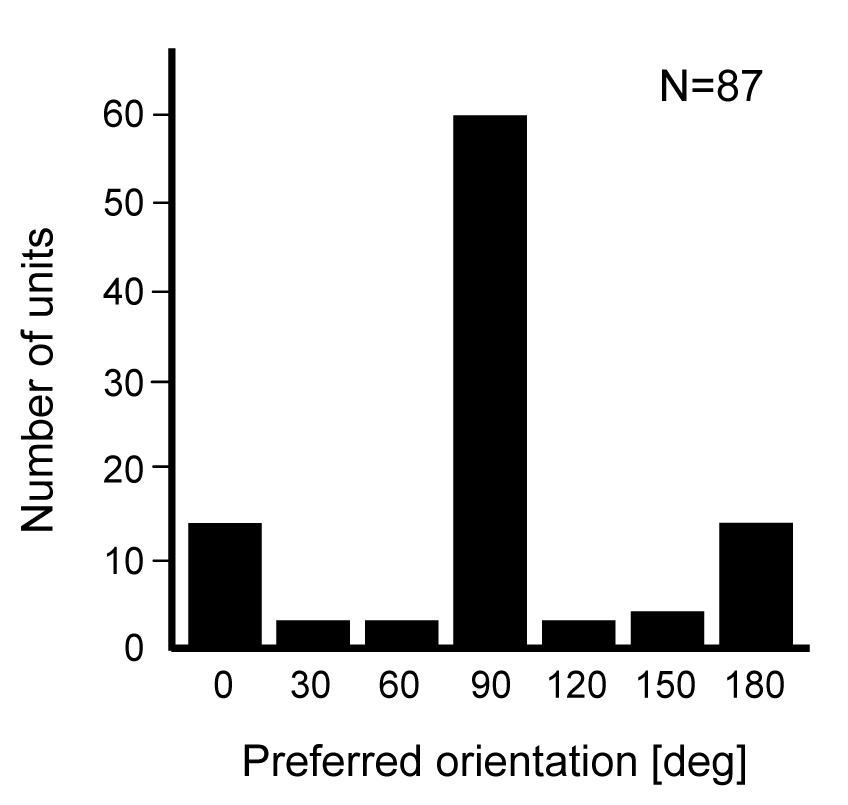

Supplement: Figure S3 — Orientation histogram obtained from single-unit recordings along the tracks when electrodes were penetrated into the medial bank of 3 cats exposed to vertical orientation. By courtesy of Dr. Ohzawa. (2.06 MB TIF) [file pone.0005380.s003.tif]

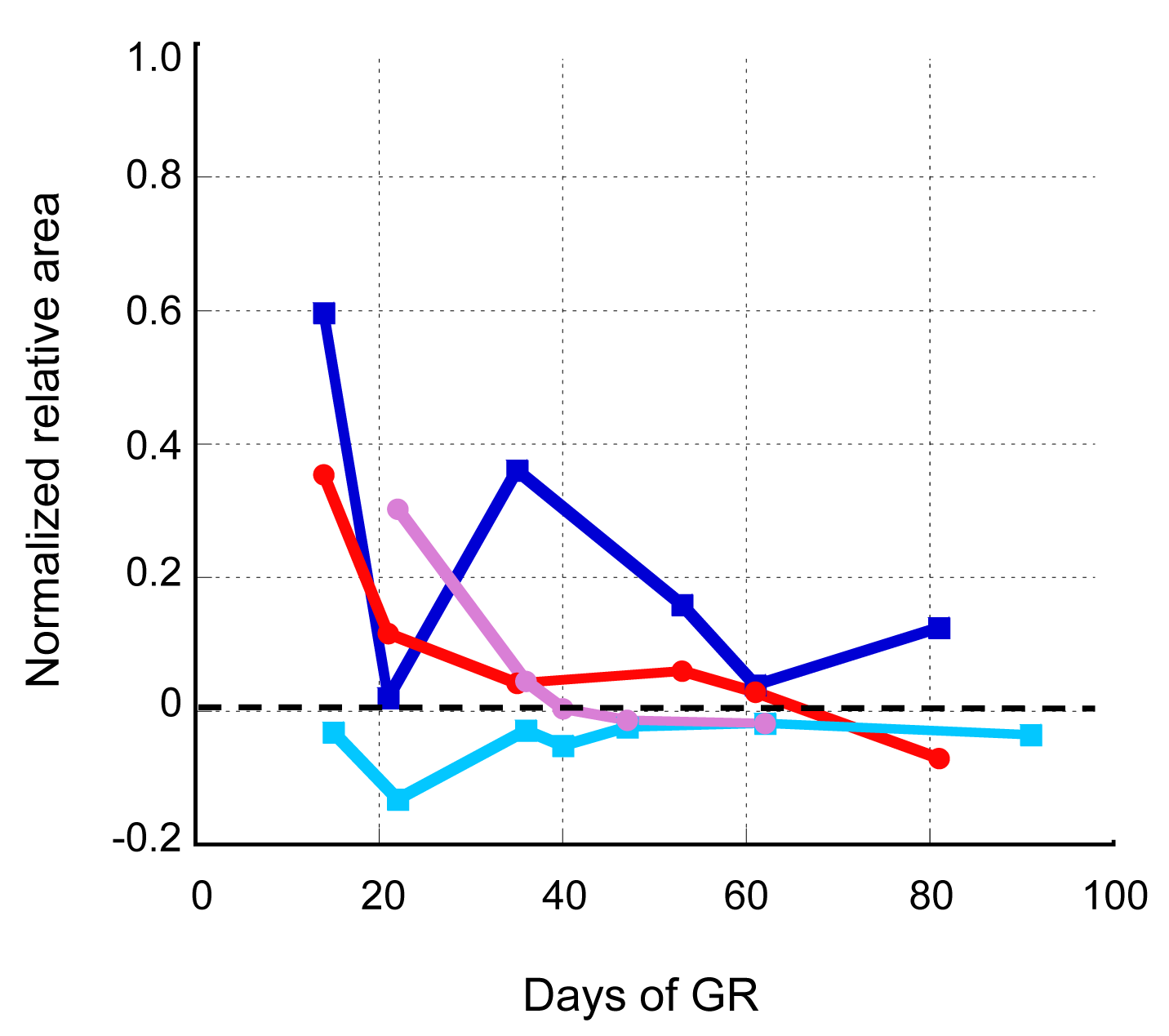

Supplement: Figure S4 — Time course of the normalized relative area of the exposed orientation in kittens exposed to a stationary stripe pattern. Blue: horizontally oriented stripe of 0.15 cpd; Red: vertically oriented strip of 0.15 cpd; Sky blue: horizontally oriented stripe of 0.5 cpd; Pink: vertically oriented stripe of 0.5 cpd. The origin in the day of GR indicates the onset day when GR was started. (5.47 MB TIF) [file pone.0005380.s004.tif]
